# Neurological sphincter deficiency: is there a place for artificial urinary sphincter?

**DOI:** 10.1007/s00345-023-04716-1

**Published:** 2024-02-03

**Authors:** Desiree Vrijens, Harry Kendall, François Hervé

**Affiliations:** 1 Departement of Urology, Maastricht University Medical Centre, Maastricht, Netherlands; 2 Departement of Urology, University Hospital of Ghent, Ghent, Belgium

**Keywords:** Neurogenic stress incontinence, Neurogenic lower urinary tract dysfunction, Artificial urinary sphincter, Sphincter deficiency

## Abstract

**Purpose:**

Neurogenic stress urinary incontinence (N-SUI) is a condition with serious impact on the quality of life. There are several treatment modalities of which the artificial urinary sphincter (AUS) stands out as the most suitable technique for addressing sphincter insufficiency. In this article, the purpose is to describe practical considerations, outcomes, and complications of the artificial urinary sphincter in neurological sphincter deficiency in both males and females.

**Methods:**

A narrative review of the current literature.

**Results:**

The outcomes of AUS are reasonably good in patients with NLUTD, the surgical technique is discussed as well as the limitations and special considerations in this complex and heterogeneous patient population.

**Conclusion:**

The available evidence suggests that its efficacy and functional durability may be lower in patients with neurogenic lower urinary tract dysfunction (NLUTD) compared to those without neurological deficits. However, studies have shown that AUS can still provide effective and safe continence outcomes in both male and female patients, with long-term device survival rates ranging from several years to over a decade.

## Introduction

Neurogenic stress urinary incontinence (N-SUI) is a condition with serious impact on the quality of life. Continence is normally achieved by a complex network of neural pathways, which include lumbosacral reflexes, spinobulbar reflexes, and periaqueductal gray and higher brain centers. In neurological disease, this pathway can be interrupted, and this may disturb the pudendal nerve which innervates the urethral sphincter and therefore result in an open bladder neck and sphincter underactivity. Lesions at the sacral or infrasacral regions, such as sacral agenesis, spina bifida, sacral spinal cord lesions, or cord compression, but also lesions involving the cauda equina and the conus medullaris, or pelvic trauma may lead to this problem [1].

Several surgical options are described to help patients suffering from N-SUI, such as sling procedures, autologous and synthetic, adjustable continence therapy, bulking agents, and artificial urinary sphincter (AUS) [2, 3]. In general, the results and complication rate of each operative technique are less favorable in patients with neurological disease compared to patients without neurological deficits. Possible reasons could be the often simultaneous presence of detrusor overactivity and lower compliance, but also inferior tissue quality is hypothesized [3].

AUS was introduced for the first time in 1972 by Foley and in 1983 Light and Scott [4] introduced the AUS for patients with neurological disorders, and it is widely used in males with neurological stress incontinence, but less in females as it is not included in current guidelines [1]. The procedure in neurogenic patients can be performed by an open, laparoscopic or robot-assisted approach as the cuff is placed around the bladder neck.

## Methods

To address the current state of the art of the AUS in neurological sphincter deficiency, we propose a narrative review. In this article, we want to describe practical considerations, outcomes and complications of the topic in both males and females. This is achieved by providing a comprehensive understanding of the existing literature regarding AUS for N-SUI patients through a qualitative analysis.

### Surgical options for N-SUI

Surgical options for the treatment of N-SUI encompass a range of techniques, each with its own advantages and limitations. Among these options, the artificial urinary sphincter (AUS) stands out as the most suitable technique for addressing sphincter insufficiency. Recently, a systematic review by Musco et al. demonstrated the efficacy and safety of surgical options for N-SUI and their findings are discussed here.[2] Musco and colleagues demonstrate that the AUS is widely used, with approximately 50 percent of surgically treated N-SUI patients receiving an AUS prosthesis. In comparison, other surgical approaches focus on increasing bladder outlet resistance. One effective method is sling placement, which can be achieved using either autologous or synthetic materials. In females, the pubovaginal sling (PVS) has shown a dry rate of 83%, while in males, a fascial sling has achieved a dry rate of 74%. Synthetic tapes, such as transobturator tapes (TOT) and retropubic transvaginal tapes (TVT), have demonstrated a continence rate of 79% in females.

However, male synthetic slings have a lower effectiveness of 50%. Another technique utilized is adjustable continence therapy, involving the placement of balloons through a perineal incision to achieve urethral compression. Unfortunately, the above-mentioned systematic review reported low continence rates of only 12% for this approach. Lastly, bulking agents have been described as a minimally invasive treatment for N-SUI, although evidence is limited to small study populations. In summary, while various surgical options exist, the AUS remains the most appropriate and effective technique for managing sphincter insufficiency in N-SUI patients, while slings, although providing improvement, may not be optimally suited for women due to their mechanism of providing extra support to the urethra.

### Outcomes - AUS in males

Few long-term follow-up studies have been conducted. Guillot-Tantay et al. [5] observed 14 males with spina bifida or spinal cord injury for more than 18 years. Out of the 14 patients, three still had their original devices in place, while eight required revisions and three had to have their devices explanted due to erosion or infection. The continence rate in this study was 50%.

It is worth noting that the efficacy and functional durability of the AUS appear to be lower in patients with neurogenic lower urinary tract dysfunction (NLUTD) compared to men without neurological deficits. Murphy et al.[6] reported that after six years, only 15% of neurogenic patients had their original device without revisions, compared to 41% of non-neurogenic patients. Additionally, the results in the neurogenic group were less favorable.

The results of the periprostatic insertion of a AUS demonstrate satisfactory long-term results, with 74% acceptable continence with a working device after 10 years of follow-up [7].

Another study compared the results of 65 patients based on the placement of the cuff and reported no significant difference about long-term explantation-free survival between bulbar urethra or bladder neck. On multivariate analysis, intermittent self-catheterization (ISC) was the only predictor of shorter survival of the device. Of the men who still had a functional device in situ 83% had satisfactory continence when the cuff was placed around the bulbar urethra after a median follow-up of 21 years and in the group where the cuff was placed around the bladder neck this was 75%, after a median follow-up of 16 years [8].

Recently, the results of robot-assisted laparoscopic AUS implantation (R-AUS) in men with neurogenic SUI have been described [9]. It proved to be a safe and efficient operation technique in the 19 included men. There were only minor complications and no conversion was needed, the continence rate was nearly 90% after a median follow-up of 58 months.

Mor et al. [10] conducted a study nearly 20 years ago, describing reasonably good results of 73% continence on clean catheterizations following augmentation cystoplasty and the insertion of a sphincter cuff alone. However, in two out of eleven patients, a pump and reservoir needed to be placed in a second stage to achieve continence. Unfortunately, two cuffs had to be removed due to erosion.

Another study, conducted by Ramsay et al. [11], reported on the placement of a cuff alone. In this single-center retrospective study with eleven patients, additional procedures such as ileocystoplasty or sphincterotomy were performed alongside the AUS placement. Six of the patients required further placement of the other two compartments of the AUS system, and all of them achieved social continence during follow-up.

### Outcomes - AUS in females

Evidence of AUS being performed in women dates back to the previous century; however, no subgroup analyses were performed to separate efficacy in men and women [4, 12]. Furthermore, literature specifically addressing the use of AUS in women with neurogenic bladder dysfunction is limited. The first retrospective study was performed in 1987, including 32 women with urinary incontinence who expressed urethral dysfunction. They report a 91% continence after AUS with one device removal due to infection and 21% mechanical complications were reported [13]. To this date, AUS is still not widely used in female patients.

Overall use of AUS in females has been proven to be effective and safe demonstrated by Costa et al. In a population of 344 female patients, there was a continence rate of 85.6% with a mean follow-up of 9.6 years. The mean device mechanical survival was 14.7 years. Safety was demonstrated by a complication rate of 26.3% with 48/99 cases being non-mechanical complications and 51/99 mechanical [14]. Ferreira et al. report an outcome of 77.6% continence when reporting on laparoscopic AUS placement in 52 female patients with a mean follow-up of 37.5 months [15]. More recently, a continence rate of 68.9% was stated in a retrospective study toward the outcome of AUS in non-neurogenic women aged 75 years or above. 71.1% of the 45 patients had their initial prosthesis after a mean follow-up of three years [16].

Long-term functional outcome of AUS in female SUI due to neurological disease is inferior to the abovementioned results. Of 26 patient who received an AMS 800 prosthesis continence was achieved in 57.7%. Two patients had their devices permanently explanted due to infection or erosion and nine patients were in need of revision surgery of which three were eventually permanently explanted due to erosion [17]. A better outcome was reported by Tricard et al. who report a 69.6% continence rate in a retrospective cohort of 33 female patients who underwent laparoscopic AUS implantation for neurological SUI between 1994 and 2014 [18]. Adherent to this a retrospective study of the charts of spina bifida patients was published in 2021 exhibiting a fully continence rate of 73.9% at last follow-up in twenty-three patients. However, this satisfactory long-term outcome was associated with high reoperation rates with a median of ten years till first reoperation. Survival rate without reoperation and without explantation after ten years were 41.8% and 66.3% respectively.[19]

### Approach

In male patients, the artificial urinary sphincter (AUS) is typically placed around the bulbar urethra using an open perineal procedure. In male patients with NLUTD, the International Continence Society (ICS) has recommended to place the cuff around the bladder neck [20]. There are several reasons for this recommendation, there is for instance a risk of perineal pressure sores in a wheelchair-based population[8]. The frequent recurrence of bladder stones and the need for endoscopic removal of the stones with an endoscope could also be a risk of erosion with a peribulbar cuff. However, the most important aspect in the neurogenic population is the need for ISC which makes [8] the risk of injury in the bulbar urethra with an increased risk of explantation [20].

The open procedure in males is very well described by Chartier-Kastler [7]. It can be a technically challenging operation. After the midline sub-umbilical incision and separation of the bladder from the peritoneum, the vas deferens is dissected and used as a guide to dissect between the seminal vesicles, to ensure that the ureters are not in the surgical field. After opening of the retropubic space and opening of the endopelvic fascia, the tape can be placed around the bladder neck under direct vison, and the placement of the balloon and the pump are done as usual.

Over the past decade, the robotic-assisted placement of the artificial urinary sphincter (AUS) has emerged as a prominent surgical procedure. This technique was first described by Yates in men with neurogenic stress incontinence [21]. They used a three-arm da Vinci® robot with a transperitoneal five-port approach. While the patients were in a 30° reverse Trendelenburg position, a posterior peritoneal incision to the seminal vesicles and bladder neck is made. When lateral prostate dissection is done and the precise location of the bladder neck on both sides is identified the cuff, after proper measurement, can be placed. Connection and placement of the tubing for the reservoir and pump is done via a small right iliac fossa incision.

As in males with N-SUI, the cuff in female patients with sphincter deficiency is placed around the bladder neck. Access to the bladder neck implies an abdominal approach and has its difficulties due to absence of a natural plane between the urethra and vagina when dissecting [22]. An open procedure is performed using the retro-pubic approach as described by Tricard et al [23] wherein the bladder neck is anteriorly approached after systematic opening of the bladder dome. Subsequently the reservoir is placed in the Retzius space and the pump inserted in the major labia. Similar to male AUS placement, advancements have been made toward laparoscopic and robot-assisted implantation in females. Laparoscopic AUS placement has been well described [24, 25] placing patient in a 30-degree Trendelenburg position using four entry points. The bladder neck is approached by incision of the parietal peritoneum and identified using a catheter. The urethra is cautiously dissected from the anterior vaginal wall and finally the cuff is placed around the urethra. Due to the technical assistance of the robotic approach, such as the magnified 3D image, tremor filtering and endowrist technology, which enhances dexterity, dissection of the bladder neck is simplified and allows more precise surgery [22]. Recently, an abstract has been published and presented by Peyronnet et al. during the 2022 ICS annual meeting describing their findings in 182 robot-assisted AUS implantations in female patients without neurological disease [26]. This approach was deemed feasible and safe as they reported a 83.5% complete continence rate with only 6.1% explantations. Before this publication, Peyronnet and colleagues also described the outcome and complications of robot-assisted AUS placement in 2018 including forty-nine female patients, including 5 patients with an underlying neurological condition. Similarly, a full continence rate of 81.6% was found at a mean of 18.5 month follow-up. One explantation was needed due to erosion accounting for 2.1% and 3 revisions were performed (6.1%). Notably there was a high intraoperative complication rate of 16.3%. [27] A systematic review comparing open and laparoscopic or robot-assisted procedures was published is 2018. They found a decrease in complication rate and explantations, respectively ranging from 5.8% to 43.8%, from 0% to 16.7%, and from 0 to 25%. Furthermore, vaginal injury rate was similar in all three groups. They report a large variety of postoperative complications between the robotic and open approach, 16.7% to 33.3% and 4.1% to 75% respectively, hypothesizing that this variety is due to outcome reporting bias. Another demonstrated difference was a lower range of explantations between open, laparoscopic and robotically assisted procedures, from 0% to 45.3%, from 0% to 8.1% and from 0% to 22.2% respectively. However, all reviewed articles were limited by a small sample size and there was a large variety in the length of follow-up between studies. An interesting finding in this review was the highest explantation rate (45.3%) being reported in the population with the largest percentage of neurogenic SUI (50%) [28].

A modification of the technique is described in a small cohort of 18 young males of which 13 (mean age of 15 years old) received a cuff-only AUS during augmentation cystoplasty, ultimately 9 patient required conversion to complete AUS. There were no complications in the cuff-only group, and after conversion to complete system, no AUS-specific complications were seen, the authors conclude that it is feasible and safe with fewer complications and may provide continence in a third of the patients and provides time for the child to mature [29].

### Limitations of AUS in patients with NLUTD

The patients who receive an AUS of course need to have a preserved manual dexterity [18]. In addition, the need for catheterization (ISC or by a caregiver) can be a risk factor for failure of the device. In general, prolonged catheterization has been proven to be a risk factor for AUS erosion in males. 44 out of 200 men used ICS, of these 17 developed erosion over an average follow-up of 24 months. [30] However, contradictive results arise between male and female spina bifida patients. As previously described, Cox regression analysis in 65 male spina bifida patients demonstrated ISC to be the only significant predictive factor for AUS survival [8]. This possible drawback for the use of AUS in the N-SUI population is backed up in a recent review report [31]. However, Gasmi et al., assessed predictors for reoperation- and explantation-free survival in 23 females with spina bifida, 69.6% of which had the need for ICS. Both ICS and wheelchair use were not statistically associated with reoperation-free survival. Wheelchair use was the only statistically significant predictive factor for explantation-free survival. These results should be interpreted witch caution due to the small cohort.[19]

Poinas describes the long-term survival of 367 AUS in female patients, 11% (42) of the patients had SUI due to neurological causes, mostly spina bifida (29 patients). In multivariate analysis, equal or more than 2 previous incontinence surgeries (RR 2.14, *p* = 0.003) and a neurological cause (RR 1.91, *p* = 0.005) was a risk factor for shorter survival of the device [32].

The procedure is more challenging in the neurological population, as often the procedure has to be combined with a bladder augmentation, when there is a low bladder compliance [2]. Furthermore, one has to take into account that when neurogenic detrusor overactivity is present and a cystoplasty is not done before or during placement of an AUS, almost 6% of the patients need a subsequent bladder augmentation [2]. A thorough pre-operative work-up and follow-up is therefore crucial.

There is considerable heterogeneity when it comes to analyzing the outcomes of surgery for incontinence in patients with NLUTD. One major contributing factor to this heterogeneity is the variation in the definition of success used across different studies [33]. The criteria for success in NLUTD surgical interventions can vary significantly from article to article. For example, some studies define success as a patient being able to use only one pad for urinary control, while others consider success as complete continence without the need for any diapers or pads at all. These varying definitions make it challenging to compare and consolidate the results of different studies as the interpretation of success can differ significantly. Therefore, when examining the surgical outcomes for NLUTD patients, it is crucial to consider the specific definition of success used in each study to accurately assess the effectiveness of the interventions.

There should be a focus on improvement of quality of life; nevertheless, the impact of interventions in patients with NLUTD on QoL is poorly described [33]. In addition, there are several studies, not related to AMS, were positive clinical results and not always associated with improved patient-reported outcome.[33]. It is important to discuss these issues with the patient before the procedure and to be aware of their expectations.

Further research, larger studies, and long-term follow-up evaluations are needed to better understand the outcomes and refine the surgical techniques in order to improve continence rates and quality of life for patients with neurogenic stress urinary incontinence.

## Conclusion

While AUS remains the most suitable technique for addressing sphincter insufficiency in both male and female patients, the available evidence suggests that its efficacy and functional durability may be lower in patients with neurogenic lower urinary tract dysfunction (NLUTD) compared to those without neurological deficits. However, studies have shown that AUS can still provide effective and safe continence outcomes in both male and female patients, with long-term device survival rates ranging from several years to over a decade.

Robotic-assisted placement of AUS has gained prominence in surgical practice during the last decade, offering enhanced precision and surgical outcomes.

Studies highlight the varied outcomes and challenges associated with the use of AUS in patients with NLUTD, emphasizing the need for careful consideration and individualized management in this population.

## Data Availability

All information used is in Pubmed, no additional data used.
